# A Digital Health Tool to Understand and Prevent Cannabis-Impaired Driving Among Youth: A Cross-sectional Study of Responses to a Brief Intervention for Cannabis Use

**DOI:** 10.2196/25583

**Published:** 2021-03-02

**Authors:** Georgina Moreno, Trevor van Mierlo

**Affiliations:** 1 Department of Economics California State University, Long Beach Long Beach, CA United States; 2 South Bay Economics LLC Hermosa Beach, CA United States; 3 Evolution Health Systems Toronto, ON Canada

**Keywords:** cannabis use, driving after cannabis use, internet, intervention, online intervention, digital health, cannabis, drug, online tool, youth, adolescent, Canada

## Abstract

**Background:**

Cannabis legalization has raised concern about an increased risk of cannabis-impaired driving, particularly among youth. Youth advocates and policy makers require cost-effective tools to target educational resources to promote responsible cannabis use.

**Objective:**

The objective of this paper is threefold. First, it describes how a youth advocacy organization disseminated a low-cost digital brief intervention to educate and inform young people about responsible cannabis use. Second, it illustrates how digital tools can help promote understanding about attitudes and behaviors toward cannabis while simultaneously offering tailored education. Finally, this paper contributes to examining behavioral factors associated with youth cannabis-impaired driving by quantifying relationships between cannabis users' willingness to drive impaired and self-reported demographic and behavioral factors.

**Methods:**

This paper analyzed data from 1110 completed Check Your Cannabis (CYC) brief interventions between March 2019 and October 2020. The CYC asks respondents a brief set of questions about their cannabis use and their personal beliefs and behaviors. Respondents receive comprehensive feedback about their cannabis use and how it compares with others. They also receive a summary of reported behaviors with brief advice. An ordered probit model was used to test relationships between cannabis use, demographics, and driving behaviors to gain further insights.

**Results:**

The vast majority (817/1110, 73.6%) of respondents reported using cannabis. However, a much smaller share of respondents reported problems associated with their cannabis use (257/1110, 23.2%) or driving after cannabis use (342/1110, 30.8%). We found statistically significant relationships between driving after cannabis use and age; Alcohol, Smoking, and Substance Involvement Screening Test (ASSIST) risk score; and polysubstance use. However, we did not find gender to be a significant determinant of driving after cannabis use. We estimated that every 10-point increase in the ASSIST score increased the probability of sometimes driving after cannabis use by 7.3% (*P*<.001). Relative to respondents who reported never drinking alcohol or using other substances with cannabis, those who sometimes drink or use other substances with cannabis were 13% (*P*<.001) more likely to sometimes or always drive after using cannabis.

**Conclusions:**

The digital health tool cost the youth advocacy organization approximately Can $0.90 (US $0.71) per use. Due to the tool's unlimited use structure, the per-use cost would further decrease with increased use by the organization’s target population. Based on our results, public health campaigns and other interventions may consider tailoring resources to frequent cannabis users, youth with high ASSIST scores, and those with polysubstance abuse. The cost-effectiveness of delivering digital brief interventions with unlimited use is attractive, as increased use decreases the per-user cost. Further research examining the efficacy of digital health interventions targeting problematic cannabis use is required.

## Introduction

### Background

The legalization of cannabis in Canada and several US states has raised concerns over cannabis-impaired driving. For example, a 2019 Gallup poll found that the top concern among opponents of legalization in the United States was the belief that legalization would increase vehicle accidents [1]. A 2019 survey found that 71% of Canadians were concerned about the impacts of cannabis legalization on road safety [2]. At the same time, many younger drivers do not perceive driving after cannabis use to be a safety risk, and some believe that cannabis use improves driving performance [3-5]. Although cannabis use impairs driving [6-11], practical and feasible means for testing for cannabis impairment are limited [8,11].

Hall [11] acknowledged that a major problem in addressing cannabis-impaired driving is the lack of a practical test for impairment and called for changing attitudes toward cannabis-impaired driving. Wadsworth and Hammond [12] compared patterns of cannabis use among youth in Canada, England, and the United States [12]. They found higher rates of driving after cannabis use among US youth relative to youth in Canada and England and attributed this finding to greater accessibility of cannabis and lower perceived harm among US youth [12]. Wadsworth and Hammond’s [12] findings suggest that understanding cannabis risk perceptions may help development of cannabis harm reduction programs.

Using an online survey of US cannabis users, Borodovsky et al [5] examined the relationship between perceived intoxication levels and driving after cannabis use and found evidence that the perception of a safe level of cannabis intoxication, not the typical level of intoxication, is associated with driving after using cannabis use. These findings support the need for identifying perceptions of harm from cannabis use and suggest that tools that can identify these perceptions may inform preventative messaging and screening for harmful cannabis use. One approach for identifying and mitigating risks associated with cannabis use is implementing a digital health screener, such as the Check Your Cannabis (CYC) brief intervention, an anonymous digital health brief intervention designed for personal computers, tablets, and smartphones [13].

The CYC, developed by Evolution Health Systems and independent academic researchers, asks respondents questions about their cannabis use and provides respondents with personalized feedback about how the severity of their cannabis use compares with others of the same age and gender. The CYC screener provides a nonjudgmental approach for educating and positively influencing respondents’ awareness of harm from cannabis consumption.

The primary theoretical behavior change constructs used in the CYC design are normative feedback, harm reduction, and motivational interviewing. The CYC’s design, workflow, and technical infrastructure are based on a similar brief intervention for addressing alcohol use, Check Your Drinking (CYD). Evolution Health Systems and independent academic researchers also developed this tool [14]. Like the CYC, the CYD provides respondents with a brief tailored feedback report summarizing personal substance use and comparing it with others of the same age, sex, and country of residence [14]. The CYD has been subject to 7 randomized controlled trials demonstrating support of its efficacy in reducing alcohol consumption [14-20].

The CYC is publicly available at no cost at a number of URLs [21,22]. The intervention consists of a 13-item questionnaire (Multimedia Appendix 1) and an output report tailored to the user (Multimedia Appendix 2). The first 3 questions of the CYC collect demographic data used to tailor the report to the individual (first name or anonymous nickname, gender, age). Questions 4 and 5 address personal use of cannabis in the past 3 months. Questions 6 to 9 address personal negative consequences of cannabis use. Question 10 addresses driving behavior following cannabis use, and question 11 addresses polysubstance abuse. Questions 12 and 13 request information about the user's cannabis expenditure patterns. Before the user submits their responses, they must actively endorse a checkbox that acknowledges that their nonpersonal information will be used for improving the tool, and they receive a link to the intervention's privacy policy.

Upon completing the questionnaire, the user receives a tailored personal report divided into either 3 or 4 sections, depending on user inputs and output algorithms. The first section contains normative feedback based on the user's age, gender, country of residence (United States or Canada), and cannabis use patterns, presented in text and graphical format. The second section reports the user's estimated annual expenditure on cannabis and compares this expenditure to purchases of movie passes and pizza slices. The third section reports the user's Alcohol, Smoking, and Substance Involvement Screening Test (ASSIST) score through text and graphical representation, which can be used to evaluate whether a person's cannabis use is problematic. If users indicate that they drive after cannabis use or use other substances with cannabis, their report includes a fourth section with information about the risks of cannabis-impaired driving, polysubstance use, or both.

In 2019, Parent Action on Drugs (PAD), an Ontario-based community youth advocacy group, licensed a white-label version of CYC from Evolution Health Systems at the standard nonprofit annual rate of Can $1000 (US $787.01), including hosting and reporting fees. Use of the tool is unlimited, meaning that there is no restriction on the number of respondents or reports generated. PAD also entered into a research partnership with Evolution Health Systems in which Evolution Health Systems assisted with the intervention dissemination strategy, data analysis, and technical guidance. PAD's white-label version of the digital intervention is available for public use on PAD’s website [23].

The primary purpose of PAD licensing a white-label version of the CYC was to offer high school students in the Greater Toronto Area information and advice about cannabis use, which Canada had recently legalized. A secondary purpose was to collect data on cannabis consumption patterns, polysubstance abuse, and driving.

### Objective

This paper analyzes the data collected from PAD’s licensed version of the CYC and focuses on the behavioral factors associated with cannabis-impaired driving from the cannabis user's perspective. The objective of this paper is threefold. First, it illustrates how low-cost brief interventions can be broadly implemented to educate young people about responsible cannabis use. Second, data from these tools can help promote understanding about attitudes and behaviors toward cannabis while anonymously offering tailored education. Finally, this paper contributes to examining behavioral factors associated with cannabis-impaired driving by quantifying relationships between cannabis users' willingness to drive impaired and self-reported demographic and behavioral factors.

## Methods

This paper's analysis relies on data obtained between March 2019 and October 2020 from the CYC brief intervention. Identical versions of the CYC intervention are available from the Evolution Health website or PAD’s websites [21-23]. PAD promoted their version of the CYC on their website and at speaking engagements at several high schools in the Greater Toronto Area. Participants were ad libitum and anonymous, and there were no incentives for completing the intervention. All study participants consented to the use of their anonymous data for research purposes. This study relied on convenience nonprobabilistic sampling, in which respondents self-selected to respond to the digital brief intervention questionnaire [24]. Multimedia Appendices 1 and 2 include images of the CYC interface.

Between March 28, 2019, and October 23, 2020, we collected 1553 completed CYC questionnaires. With the goal of informing responsible cannabis use among young adults in Canada, we limited the sample to participants aged 25 years and younger from Canada, thus reducing the sample to 1175. We eliminated from the analysis 34 responses that appeared to be duplicates. We further reduced the sample by dropping from the analysis 6 responses with extreme outlier values for the reported expenditures on cannabis and 25 responses with inconsistencies (eg, reported not using cannabis but reported positive expenditures on cannabis). The final study data set included 1110 responses. Table 1 summarizes the age and gender distribution for the included responses.

**Table 1:** Age and gender distribution for included respondents.

Gender	Minimum age (years)	Median age (years)	Maximum age (years)	Observations, n
Female	12	17	25	447
Male	12	17	25	593
Transgender or nonbinary	14	16	25	50
Not reported	14	16	18	20
Overall	12	17	25	1110

Data were extracted from the intervention platform's precoded custom structure query language database. All responses were anonymous, and data collection procedures adhered to American and Canadian privacy guidelines [25-27]. Respondents actively endorsed a checkbox consenting to the use of their nonpersonal responses to analyze and improve the intervention and received a link to the intervention's privacy policy. As the study relies on unidentifiable archival data, the study was exempt from further review.

After reporting gender and age, respondents answered 8 questions related to their use of cannabis. The questionnaire allowed respondents to report their gender as female, male, transgender, nonbinary, or not reported. Respondents also reported age as an open-ended question. The brief intervention asks respondents about their expenditures on cannabis and measures expenditures in 2 ways: the average monthly expenditure on cannabis over the past year and the largest single-day expenditure over the past year. Average monthly expenditures capture typical use. Expenditures are reported in Canadian dollars.

**Figure 1:**
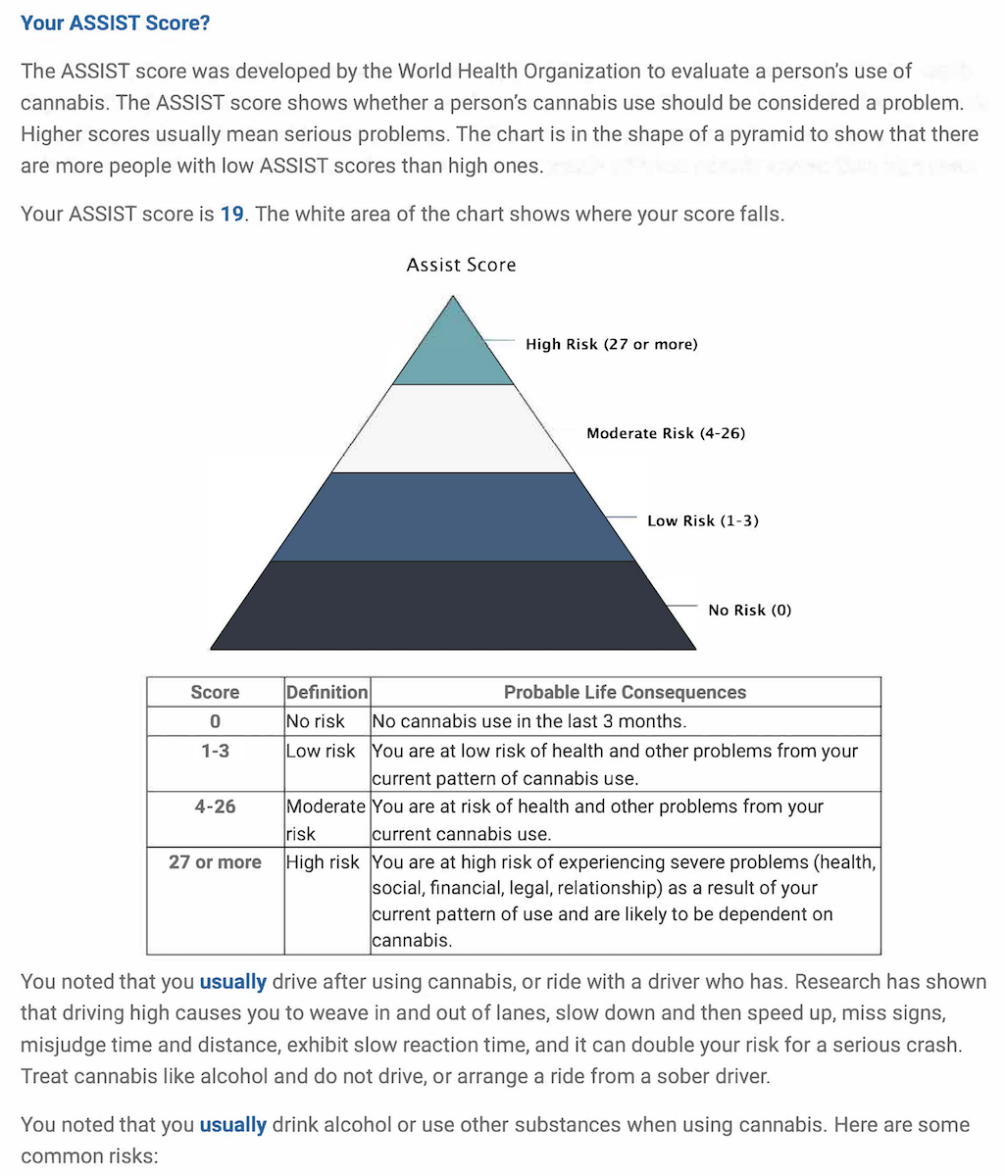
Check Your Cannabis ASSIST score. ASSIST: Alcohol, Smoking, and Substance Involvement Screening Test.

A key component of the intervention is a calculated score from the 6-item ASSIST developed by the World Health Organization (WHO) [28]. The ASSIST evaluates an individual's use of cannabis and classifies individuals into risk categories, with high risk indicating the experience of problems resulting from cannabis consumption. Figure 1 is an illustration explaining the ASSIST score after completion of the CYC questionnaire. The WHO ASSIST score is the sum of weights assigned to the 6 questions listed in Table 2 [28]. The questionnaire asks respondents to answer the 6 ASSIST questions. We computed the ASSIST score based on the response weights, also shown in Table 2.

**Table 2:** ASSIST score questions, possible responses, and WHO weights [28]. ASSIST: Alcohol, Smoking, and Substance Involvement Screening Test; WHO: World Health Organization.

Question	Response (WHO weight)
1. During the past 3 months, how often have you used cannabis?	Never (0); once or twice (4); monthly (5); weekly (6); daily or almost daily (7)
2. During the past 3 months, how often have you had a strong desire or urge to use cannabis?	Never (0); once or twice (4); monthly (5); weekly (6); daily or almost daily (7)
3. During the past 3 months, how often has your use of cannabis led to health, social, legal, or financial problems?	Never (0); once or twice (4); monthly (5); weekly (6); daily or almost daily (7)
4. During the past 3 months, how often have you failed to do what was normally expected of you because of your use of cannabis?	Never (0); once or twice (4); monthly (5); weekly (6); daily or almost daily (7)
5. Has a friend or relative or anyone else ever expressed concern about your use of cannabis?	No, never (0); yes, but not in the past 3 months (3); yes, in the past 3 months (6)
6. Have you ever tried and failed to control, cut down, or stop using cannabis?	No, never (0); yes, but not in the past 3 months (3); yes, in the past 3 months (6)

Respondents also answered questions about driving after using cannabis and polysubstance use. These questions were “How often do you drive after using cannabis, or ride with someone who has?” and “When you use cannabis, how often do you drink alcohol or use other substances?” The possible responses for these questions were “Never,” “Sometimes,” “Usually,” or “Always.”

To better understand the factors associated with driving after cannabis use, we modeled driving after cannabis use as a function of age, gender, ASSIST score, whether the respondent drinks or uses other substances with cannabis (ie, polysubstance use), and cannabis expenditures. Given the qualitative and ordered nature of the responses to the question about driving after cannabis use, we estimated the probability of driving after using cannabis using an ordered probit regression [29]. We combined the “usually drives” and “always drives” categories for the ordered probit model. Therefore, the dependent variable in the model included 3 categories: (1) never drives after using cannabis, (2) sometimes drives after using cannabis, and (3) usually or always drives after using cannabis. For this analysis, we limited the data to respondents of driving age (n=913). All analysis was conducted using Stata 16 (StataCorp) [30].

We hypothesized that age has an increasing and diminishing effect on driving after using cannabis and included age as a quadratic in the ordered probit model. Because the vast majority of participants in the analytical data reported their gender as female (447/1110, 40.3%) or male (593/1110, 53.4%), we defined the gender variable in the model as female, male, or other. Based on past studies, we expected differences by gender in cannabis use and attitudes [31]. We also hypothesized that respondents with higher ASSIST scores are more likely to drive after using cannabis, since a higher ASSIST score indicates a higher risk of harmful cannabis use. We hypothesized that polysubstance use is positively correlated with driving after cannabis use, as it reflects risky behaviors. To the extent that higher expenditures reflect greater use, we hypothesized that higher expenditures are associated with heavier cannabis use and therefore a higher probability of driving after using cannabis.

## Results

**Figure 2:**
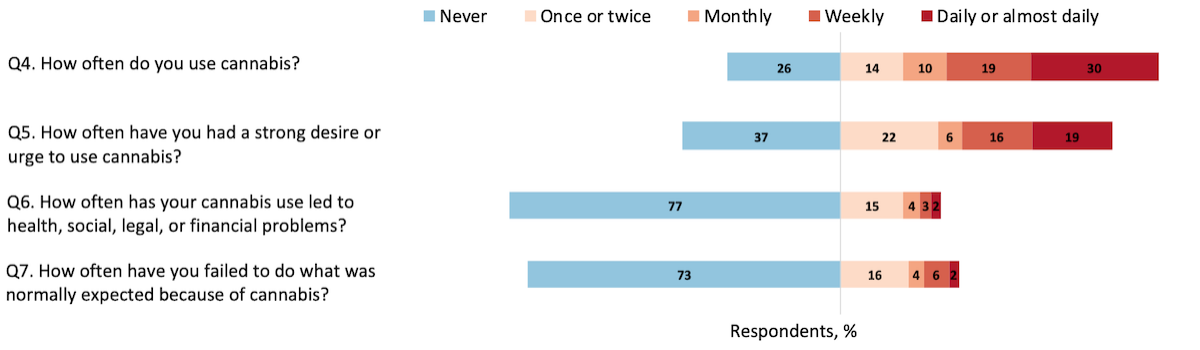
Percentage of respondents aged 12 to 25 years who reported cannabis use and problems in the past 3 months (N=1110).

**Figure 3:**
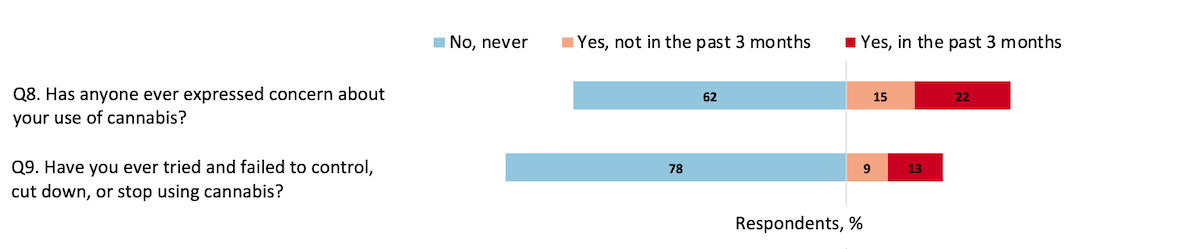
Percentage of respondents aged 12 to 25 years who reported concerns about their cannabis use (N=1110).

**Figure 4:**
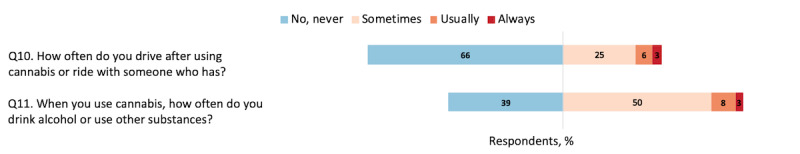
Percentage of driving-age respondents aged 16 to 25 years who reported risky behaviors (n=913).

Figures 2-4 report the distribution of responses for the 3 categories of questions included in the brief intervention: (1) cannabis use behaviors in the past 3 months, (2) concerns about cannabis use, and (3) driving after cannabis use and polysubstance use. While 73.6% (817/1110) of respondents reported using cannabis and 63.1% (700/1110) reported having a desire or urge to use cannabis, over 76.8% (853/1110) reported never having problems with cannabis use (Figure 2, question 6) and 72.7% (807/1110) reported never failing to meet expectations because of their cannabis use (Figure 2, question 7). However, among the heaviest cannabis users or those using daily or almost daily (n=330), 48.8% (161/330) reported having incidents of health, social, legal, or financial problems associated with cannabis use.

As seen in Figure 3, 22.0% (244/1110) of respondents reported having problems controlling their cannabis use (question 9). Although only 12.6% (140/1110) reported having such problems in the past 3 months, 79.3% (111/140) of the respondents in this category were aged between 12 and 18 years, below the legal age for cannabis consumption in Canada. When asked about others' concerns over their cannabis use (question 8), a smaller proportion, 62.4% (693/1110), reported “never.” Figure 4 shows that the vast majority of driving-age respondents, 66.5% (607/913), reported never driving after using cannabis (question 10), and 61.0% (557/913) reported using alcohol and other substances with cannabis (question 11).

In our sample, the ASSIST score varied from 0 to 40, with 66.3% (736/1110) of respondents falling in the moderate risk category. The average ASSIST score was 12 among teenaged respondents (aged 12-18 years) and 18 for young adult respondents (aged 19-25 years). ASSIST scores for teenagers were statistically significantly smaller than ASSIST scores for young adults (*P*<.001).

Table 3 reports the ordered probit coefficient estimates. Male gender (*P*=.28), other gender (*P*=.36), and monthly expenditures (*P*=.07) were not statistically significant at the .05 level. Age (*P*<.001), polysubstance use sometimes (*P*<.001) and usually or always (*P*<.001), ASSIST score (*P*<.001), and maximum expenditure (*P*=.02) were statistically significant.

**Table 3:** Ordered probit estimates for the question “How often do you drive after using cannabis or ride with someone who has?” (n=913).

Covariate			Coefficient estimate^a^		*P* value
Age^b^			0.6586		.04
Age^2^			–0.0162		.051
**Gender (base=female)**					
	Male	0.1105		.23	
	Other gender	–0.2876		.20	
ASSIST^c^ score			0.0450		<.001
**Polysubstance use (base=never)**					
	Sometimes	0.4221		<.001	
	Usually or always	0.5942		<.001	
Average monthly expenditure			0.0006		.11
Maximum single expenditure			0.0004		.11

^a^The log-likelihood for this model was –640.1 and the pseudo-R^2^ was 0.1569.

^b^Age and Age^2^ are jointly significant with a *P* value of .10.

^c^ASSIST: Alcohol, Smoking, and Substance Involvement Screening Test.

Table 4 reports the average marginal effects for each of the outcomes of the driving after cannabis use variable. In Table 4, ASSIST scores and polysubstance use have the largest association with driving after using cannabis. The model predicts that every 10-point increase in the ASSIST score increases the probability of sometimes driving after cannabis use by 7.3% (*P*<.001) and increases the probability of usually or always driving after using cannabis by 5.8% (*P*<.001).

Relative to respondents who reported never using other substances with cannabis, respondents who reported sometimes using other substances with cannabis were 13% (*P*<.001) more likely to report driving after using cannabis. Those who reported usually or always using other substances were 18% (*P*<.001) more likely to report driving after using cannabis. This suggests that using alcohol and other substances with cannabis increases the probability of driving after cannabis use, consistent with other studies in the literature [8].

**Table 4:** Average marginal effects for ordered probit outcomes (n=913).

Covariate			“How often do you drive after using cannabis or ride with someone who has?”									
			Never				Sometimes			Usually or always		
			Effect		*P* value		Effect	*P* value		Effect		*P* value
Age^a^			–0.0253		.03		0.0152	.03		0.0101		.03
**Gender (base=female)**												
	Male	–0.0327		.23		0.0182		.23	0.0144		.22	
	Other gender	0.0781		.17		–0.0482		.20	–0.0299		.14	
ASSIST^b^ score			–0.0132		<.001		0.0073	<.001		0.0058		<.001
**Polysubstance use (base=never)**												
	Sometimes	–0.1245		<.001		0.0767		<.001	0.0478		<.001	
	Usually or always	–0.1811		<.001		0.1062		<.001	0.0750		.001	
Average monthly cannabis expenditure			–0.0002		.11		0.0001	.11		0.0001		.11
Maximum single cannabis expenditure			–0.0001		.11		0.0001	.11		0.0001		.11

^a^The marginal effect on age includes the full quadratic effect of age on the probability of driving after cannabis use.

^b^ASSIST: Alcohol, Smoking, and Substance Involvement Screening Test.

**Figure 5:**
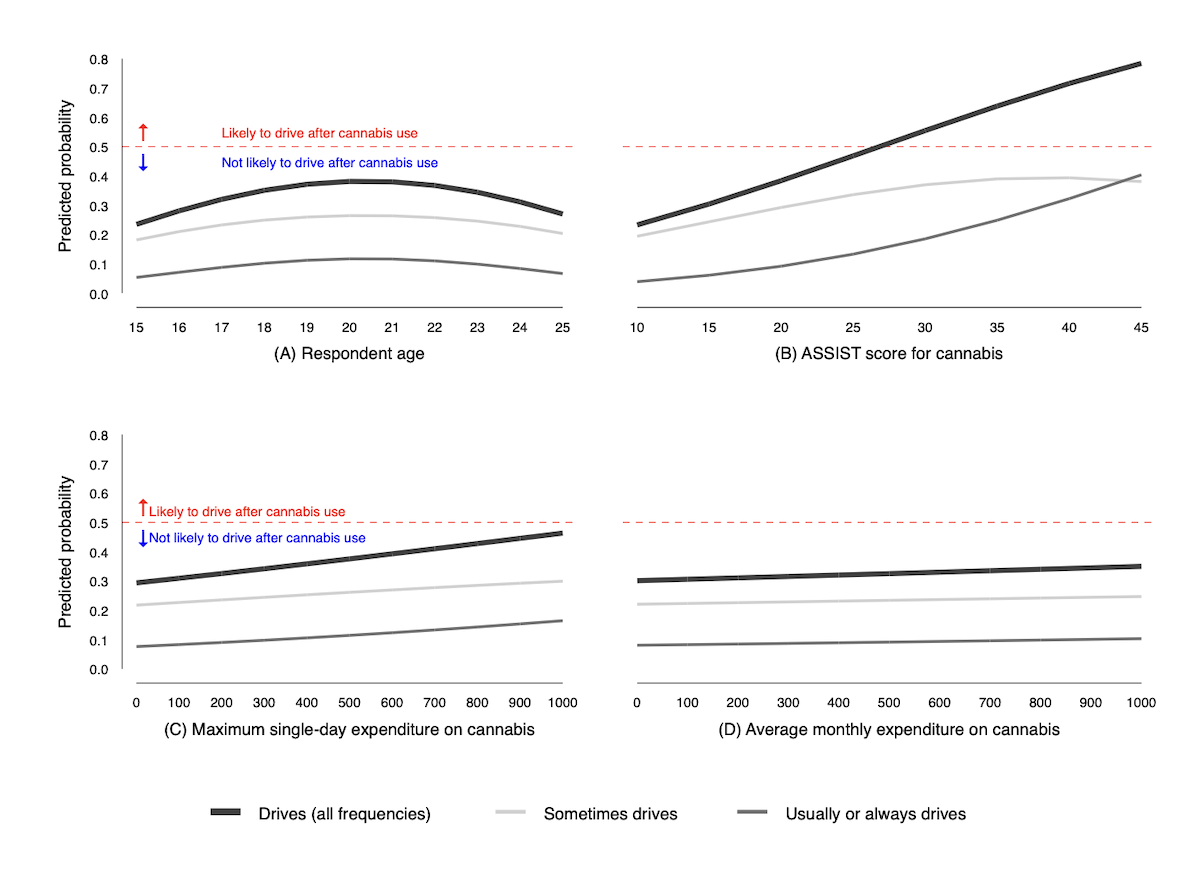
Probability of driving after cannabis use is low over a range of respondent characteristics (n=913). ASSIST: Alcohol, Smoking, and Substance Involvement Screening Test.

Figure 5 plots the predicted probability of driving after using cannabis at varying levels of age, ASSIST scores, maximum expenditures, and average expenditures. This figure plots the predicted probability by respondents who sometimes drive and those who usually or always drive after cannabis use and the overall predicted probability of driving after cannabis use (ie, the probability of sometimes driving plus the probability of usually or always driving). In Figure 5, we explored how the predicted probability of driving after cannabis use changes with respondent characteristics. If the predicted probability exceeds 0.5, the model predicts that respondents are more likely than not to drive after cannabis use, indicated with a red dashed line in the figure.

Panel A of Figure 5 shows that holding constant gender, ASSIST score, and cannabis expenditures, the overall probability of driving after cannabis use was 0.28 for the youngest respondents in the sample, peaked at 0.35 for 20-year-olds, and dropped to approximately 0.20 for the oldest respondents. Panel B shows that the overall predicted probability of driving after using cannabis, all else constant, increased with ASSIST score, suggesting that respondents with ASSIST scores greater than 25 are more likely than not to drive after using cannabis. As seen in panel C, the predicted probability of driving after cannabis use increased with maximum single-day expenditures. However, the model predicted that even the respondents with the highest expenditures, those most likely to be the heaviest users, are unlikely to drive after cannabis use (ie, the predicted probability of driving after cannabis use is less than 0.5). Similarly, panel D shows that the predicted probability of driving after cannabis use increased with average monthly expenditures, holding all other factors constant. Only at extreme monthly expenditures (over Can $800 [US $630.09]) did the model predict that respondents are more likely than not to drive after cannabis use.

**Figure 6:**
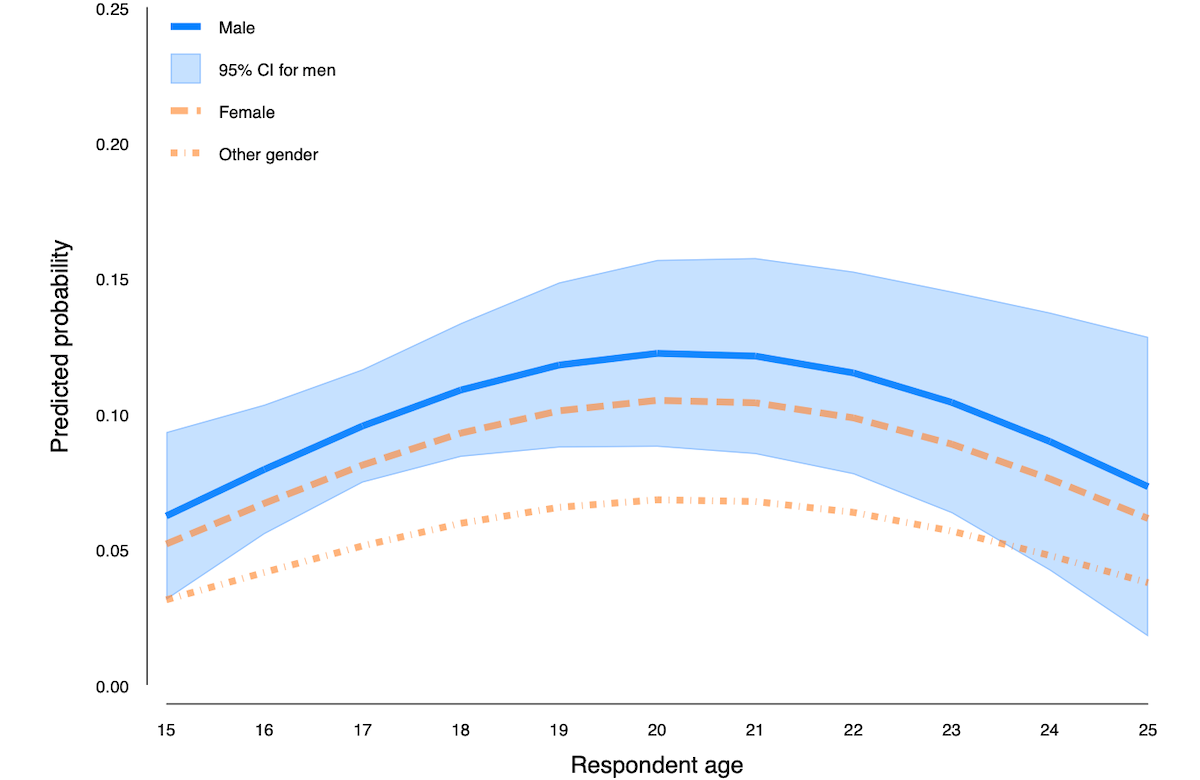
Probability of reporting usually or always driving after cannabis does not differ by gender (n=913).

Figure 6 plots the probability of responding “usually” or “always” drives after cannabis use by gender. We found that gender was not a statistically significant factor in determining the probability of driving after using cannabis after controlling for age, ASSIST score, polysubstance use, and expenditures on cannabis. The figure plots the 95% CI for the probability of usually or always driving after cannabis use for male respondents and shows that the probability of usually or always driving after cannabis use for female respondents is within the 95% CI for men at all ages. Respondents younger than 24 years who reported being of another gender have a slightly lower predicted probability of usually or always driving after cannabis use than male respondents.

Our results show that except for individuals with high ASSIST scores, after controlling for age, gender, and polysubstance use, respondents in our sample were unlikely to drive after using cannabis. Contrary to the literature [31,32], we found that gender is not a statistically significant factor in determining the probability of driving after using cannabis. We also found that polysubstance use and ASSIST scores were strongly correlated with driving after using cannabis. While the level of cannabis expenditures was statistically significant, the effects on driving after cannabis use were small.

## Discussion

### Principal Findings

The brief intervention questionnaire sheds light on self-reported harmful cannabis use behaviors. We observed that the vast majority of respondents reported cannabis use; however, they also reported not experiencing problems with cannabis use. However, daily cannabis users did report experiencing issues resulting from their use of cannabis. More respondents reported having family or others express concerns about their cannabis use than reported their own concerns about cannabis use. This may suggest that while respondents may not acknowledge problems with their cannabis use, they report an awareness of others' concerns about their use of cannabis.

At an implementation cost of Can $1000 (US $787.62), the digital health tool cost PAD approximately Can $0.90 (US $0.71) per use. Due to the unlimited use structure, the per-use cost would further decrease with PAD’s target population's increased use. The digital brief intervention is an example of a low-cost tool that public health campaigns can leverage to tailor resources toward the most at-risk populations. While we focused on cannabis-impaired driving concerns, the tool is flexible and customizable to explore specific cannabis use concerns.

### Strengths and Limitations

This study provides insights into how young cannabis users perceive their cannabis use and examines the relationship between cannabis use and driving behaviors from their perspectives. A particular strength is that the CYC intervention has limited use barriers, is anonymous, and was disseminated by a well-known youth advocacy group (PAD). However, as is common with self-reported digital health interventions, there is no way to guarantee that user responses are accurate or honest. Nevertheless, we have shown that a simple, low-cost digital health tool such as the CYC can provide insights to guide cannabis education programs and assist policy makers and youth health advocates targeting efforts for preventing cannabis abuse specific to a community.

The population analyzed in this study can be described as self-seeking and may be problematic cannabis users who actively sought help or more information on their behavior. As a result, they may not be considered representative of the general population. However, if this is the case, it may add strength to our principal finding that the largest amount spent on any given day, higher ASSIST scores, and polysubstance use were positively and significantly associated with driving under the influence of cannabis. As a result, these preliminary results should be interpreted with caution, and there is a need for replication studies that may or may not confirm our results.

### Future Research Directions

The CYC has recently been enhanced to include questions addressing the source of cannabis acquisition (primarily dispensaries or licensed services versus informal connections) as well as employment status and the user's geographic location. These additional questions are designed to generate further insight into cannabis use patterns and behavior. Geospatial analysis will allow us to gather further insights and compare use patterns across specific jurisdictions. The CYC has also been modified to include users’ first 3 characters in their zip or postal code, and ongoing research is focusing on geospatial analysis to help assess regional patterns.

Based on the ability of digital health interventions to collect self-reported demographic and behavioral data and compare and contrast these data for specific geographic areas, there is the potential for these tools to examine associations between cannabis use and driving at a regional level. This may give further insight on potential predictors of increased risk, which could support or validate findings from other, nondigital studies.

### Conclusion

To our knowledge, this is the first study to examine associations between self-reported cannabis use and driving behaviors through the use of a digital brief intervention, which has the dual purpose of educating cannabis users and collecting data to help inform and shape responsible cannabis use programs. Our analysis indicates that the largest amount spent on any given day, higher ASSIST scores, age, and polysubstance use were positively and significantly associated with driving under the influence of cannabis. Gender was not a significant factor.

We have shown how a low-cost digital health tool can inform programs and policies for educating young people about responsible cannabis use. Based on these results, public health campaigns or other interventions may have a more significant impact if they focus resources on problematic cannabis users rather than the general population. The largest amount spent variable may give insight into those who purchase their cannabis from nonretail sources. Further research is required.

## Appendix

Multimedia Appendix 1 CYC Interface.

Multimedia Appendix 2 CYC Sample Report.

## Abbreviations

ASSIST: Alcohol, Smoking, and Substance Involvement Screening Test
CYC: Control Your Cannabis
CYD: Control Your Drinking
PAD: Parent Action on Drugs
WHO: World Health Organization
