# Supplementary material for: A Digital Health Tool to Understand and Prevent Cannabis-Impaired Driving Among Youth: A Cross-sectional Study of Responses to a Brief Intervention for Cannabis Use
Source: JMIR Form Res. 2021 Mar 2;5(3):e25583. doi: 10.2196/25583 (PMC7967219; doi:10.2196/25583)
Supplement: Multimedia Appendix 1 [file formative_v5i3e25583_app1.pdf]

Questions 1-16

0% Complate

Ever taken a step back to think about how using cannabis is fitting into your life? Take this short, anonymous quiz. The answers might surprise you.

1. Your First Name (to protect your privacy please only use your first name or a nickname):

2. You are:

- ◯ Male
- ◯ Female
- ◯ Transgender
- ◯ Non-Binary / Third Gender
- ◯ Prefer not to say

3. Your age: (example: 18)

4. During the past 3 months, how often have you used cannabis?

- ◯ Never
- ◯ Once or twice
- ◯ Monthly
- ◯ Weekly
- ◯ Daily or almost daily

5. During the past 3 months, how often have you had a strong desire or urge to use cannabis?

- ◯ Never
- ◯ Once or twice
- ◯ Monthly

- ◯ Weekly
- ◯ Daily or almost daily

6. During the past 3 months, how often has your use of cannabis led to health, social, legal or financial problems?

- ◯ Never
- ◯ Once or twice
- ◯ Monthly
- ◯ Weekly
- ◯ Daily or almost daily

7. During the past 3 months, how often have you failed to do what was normally expected of you because of your use of cannabis?

- ◯ Never
- ◯ Once or twice
- ◯ Monthly
- ◯ Weekly
- ◯ Daily or almost daily

8. Has a friend or relative or anyone else ever expressed concern about your use of cannabis?

- ◯ No, never
- ◯ Yes, but not in the past 3 months
- ◯ Yes, in the past 3 months

9. Have you ever tried and failed to control, cut down or stop using cannabis?

- ◯ No, never
- ◯ Yes, but not in the past 3 months
- ◯ Yes, in the past 3 months

10. How often do you drive after using cannabis, or ride with a driver who has?

- ◯ Never
- ◯ Sometimes
- ◯ Usually

- ◯ Always

11. When you use cannabis, how often do you drink alcohol or use other substances?

- ◯ Never
- ◯ Sometimes
- ◯ Usually
- ◯ Always

12. In the **past 12 months**, what is the largest amount you estimate you have spent on cannabis, **on any given day**? (example: 10.00)

$ [ ]

13. In the **average month**, about how much money would you estimate that you spend on cannabis? (example: 50.00)

$ [ ]

14. How do you get your cannabis? Please select all that apply:

- ☐ a dispensary or licensed service
- ☐ informally from a connection
- ☐ a friend or someone else gives it to me

15. What is your employment status?

- ◯ I'm a full-time student
- ◯ I'm a part-time student
- ◯ I work part time
- ◯ I work full time
- ◯ I'm not employed, I'm retired, or I'm not able to work

16. Please enter the first three characters of your postal code (Canada) or your zip code (USA):

[ ]

☐ Please check this box to acknowledge that your non-personal information will be used for purposes of improving this program. We do not collect or analyze individual statistics and no one will contact you at any time. Please review our Privacy Policy.

Evolution Health is not a healthcare provider and does not provide medical advice, diagnosis, or treatment. If you are currently thinking about or planning to harm yourself or someone else please call 911 or go to the nearest hospital emergency room.

© Copyright 2020 Evolution Health Systems. All Rights Reserved.
