# Supplementary material for: A Digital Health Tool to Understand and Prevent Cannabis-Impaired Driving Among Youth: A Cross-sectional Study of Responses to a Brief Intervention for Cannabis Use
Source: JMIR Form Res. 2021 Mar 2;5(3):e25583. doi: 10.2196/25583 (PMC7967219; doi:10.2196/25583)
Supplement: Multimedia Appendix 2 [file formative_v5i3e25583_app2.pdf]

Send To Email | Print

Check Your Cannabis Results for Trevor

About **60.5%** of **46** year old American men have used cannabis at least once in their lifetime. This pie chart shows how often 46 year old American men have used cannabis ever, or in the last year.

The highlighted area is where your cannabis use fits in:

Cannabis Use 46 Year Old American Men

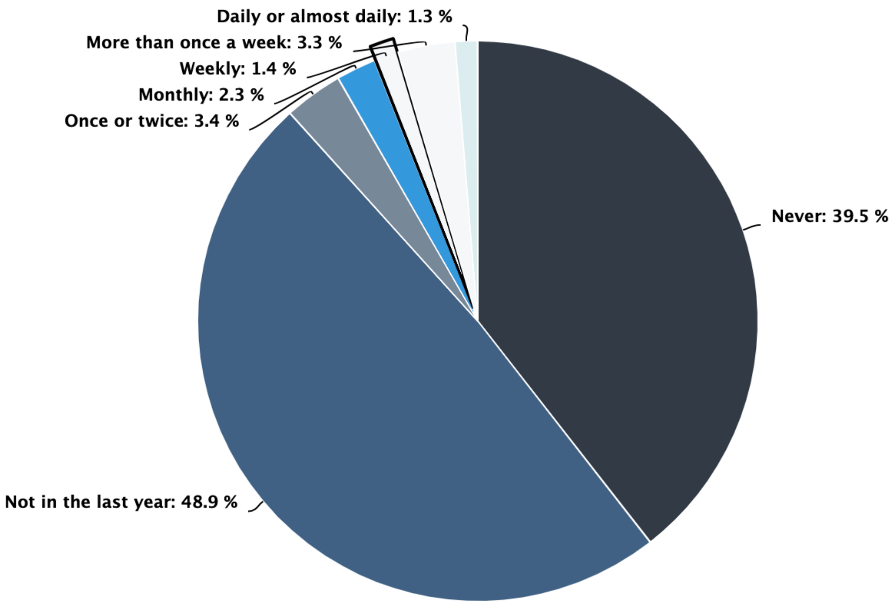

You use cannabis more often than **94.1%** of 46 year old American men.

How Much Do I Spend?

Based on the information you gave us, you spend approximately **$75.00** per month, or approximately **$900.00** per year on cannabis. The largest amount you reported spending on any given day in the past 12 months is **$10.00**.

With this money you could have bought **100** movie passes:

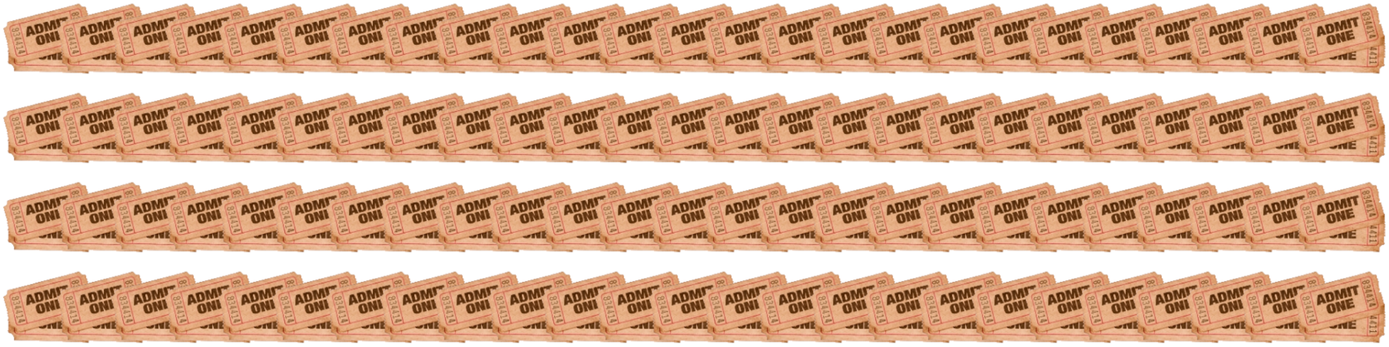

or **360** slices of pizza.

Your ASSIST Score?

The Alcohol, Smoking and Substance Abuse Scoring (ASSIST) Test was developed by the World Health Organization (WHO) to evaluate a person's use of cannabis. The ASSIST score shows whether a person's cannabis use should be considered a problem. Higher scores usually mean serious problems. The chart is in the shape of a pyramid to show that there are more people with low ASSIST scores than high ones.

Your ASSIST score is **19**. The white area of the chart shows where your score falls.

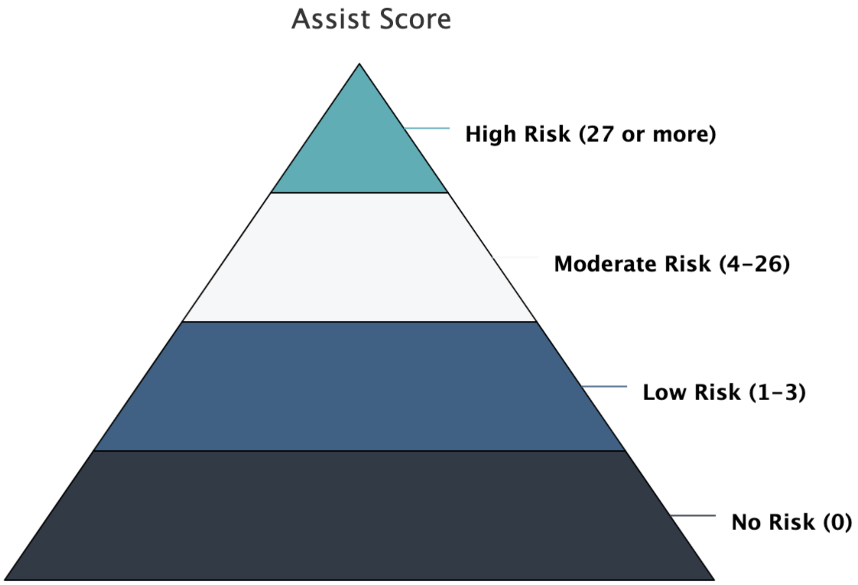

Score	Definition	Probable Life Consequences
0	No risk	No cannabis use in the last 3 month.
1 - 3	Low risk	You are at low risk of health and other problems from your current pattern of cannabis use.
4 - 26	Moderate risk	You are at risk of health and other problems from your current cannabis use.
27 or more	High risk	You are at high risk of experiencing severe problems (health, social, financial, legal, relationship) as a result of your current pattern of use and are likely to be dependent on cannabis.

You noted that you **usually** drive after using cannabis, or ride with a driver who has. Research has shown that driving high causes you to weave in and out of lanes, slow down and then speed up, miss signs, misjudge time and distance, exhibit slow reaction time, and it can double your risk for a serious crash. Treat cannabis like alcohol and do not drive, or arrange a ride from a sober driver.

You noted that you **usually** drink alcohol or use other substances when using cannabis. Here are some common risks:

Greening Out: Drinking alcohol or using other substances before consuming cannabis can increase the likelihood of greening out - feeling nauseous, dizzy, needing to lie down, and vomiting.

Intensified Effects: Highs experienced from cannabis, alcohol, or other substances can be felt sooner and more intensely when used together. When drinking before using cannabis, higher levels of THC are absorbed. This often results in an increased heart rate, memory impairment, poor judgement, or problems negotiating consensual or safer sex.

Overuse: Combined use of substances increases all kinds of risks, including passing out or making bad decisions. Vomiting is how your body gets rid of excess alcohol or reacts when using more than one substance. Cannabis makes it difficult to vomit which increases your risk of choking and/or alcohol poisoning.

You mentioned that you get your cannabis **from a dispensary or licensed service**. That's the safest bet. If you get your cannabis from another source, you can never be sure what you're buying.

Back To Questions

Clear Data
